# New therapeutic approaches of mesenchymal stem cells-derived exosomes

**DOI:** 10.1186/s12929-021-00736-4

**Published:** 2021-05-25

**Authors:** Jana Janockova, Lucia Slovinska, Denisa Harvanova, Timea Spakova, Jan Rosocha

**Affiliations:** Associated Tissue Bank, Faculty of Medicine, P. J. Safarik University in Kosice, Tr. SNP 1, 04011 Kosice, Slovakia

**Keywords:** Mesenchymal stem cells, Exosomes, Cell-free therapy, Therapeutic potential

## Abstract

Mesenchymal stem cells (MSCs) have been demonstrated to have a great potential in the treatment of several diseases due to their differentiation and immunomodulatory capabilities and their ability to be easily cultured and manipulated. Recent investigations revealed that their therapeutic effect is largely mediated by the secretion of paracrine factors including exosomes. Exosomes reflect biophysical features of MSCs and are considered more effective than MSCs themselves. Alternative approaches based on MSC-derived exosomes can offer appreciable promise in overcoming the limitations and practical challenges observed in cell-based therapy. Furthermore, MSC-derived exosomes may provide a potent therapeutic strategy for various diseases and are promising candidates for cell-based and cell-free regenerative medicine. This review briefly summarizes the development of MSCs as a treatment for human diseases as well as describes our current knowledge about exosomes: their biogenesis and molecular composition, and how they exert their effects on target cells. Particularly, the therapeutic potential of MSC-derived exosomes in experimental models and recent clinical trials to evaluate their safety and efficacy are summarized in this study. Overall, this paper provides a current overview of exosomes as a new cell-free therapeutic agent.

## Background

Nowadays, multipotent mesenchymal stem cells (MSCs) have been extensively examined because of their usage in clinical trials. Their effective influence in cellular therapy and regenerative medicine is known for their strong immunosuppressive, immunomodulatory and regenerative activity [1, 2]. In addition, their considerable potential was demonstrated in the treatment of immune-mediated, inflammatory and degenerative diseases [3–9].

**Fig. 1:**
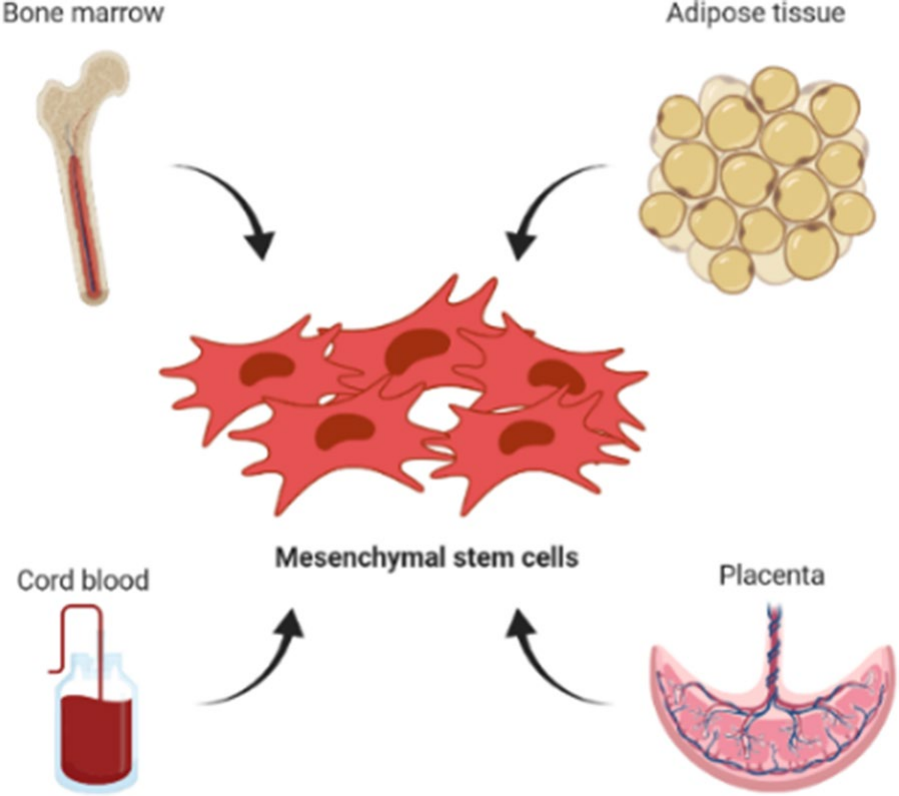
The most common sources of MSC isolation. (Created with BioRender.com.)

MSCs generally are multipotent, somatic progenitor/stem cells first isolated from adult bone marrow [10, 11] and successfully differentiated from marrow hematopoietic cells according to their adherent nature in in vitro cell lines and fibroblastic morphology. They are able to self-recover and retain variable differentiation potency toward multi-lineages [12, 13]. The International Society for Cellular Therapy has officialy defined minimal criteria for MSCs, following as (a) being plastic-adherent cells, (b) having adipogenic, osteogenic and chondrogenic trilineage mesenchymal differentiation capacity and (c) being positive (> 95%) for surface antigens CD73, CD90 and CD105 and negative (< 2%) for hematopoietic markers CD34, CD45, CD14 or CD11b, CD79α or CD19 and HLA-DR (typical markers of hematopoietic cells) [14]. Human MSCs were described in many tissues (Fig. 1), not only in those of mesodermal origin (bone marrow, bone, adipose, synovial membrane and muscle) but also in skin, heart, lungs, brain, kidneys, thymus, liver and pancreas [14, 15]. Another excellent sources of human MCSs are umbilical cord tissue and placenta [16–18]. However, it was revealed that MSCs obtained from various tissues have differences in gene expression, proliferation activity and differentiation potencial. In addition, some variations in surface antigens expression compared to requirements of minimal criteria were reported. Existing variances indicate specific features of MSCs from different tissues and organs or are related with isolation and cultivation protocols [19]. MSCs from different tissues can be cultured prior to clinical use. They can grow easily in the culture dish which leads to an easy manipulation in terms of isolation and cultivation. Subsequently, prepared MSCs suspensions may be introduced intravenously or trough local injection to obtain the required therapeutic effects directly or indirectly [20]. Further characteristics include typical plasticity, intrinsic tropism towards injured or inflammed area (known as homing) and an extensive release of numerous useful growth factors, cytokines and another bioactive soluble factors as important indication of their potential clinical applications in tissue repair and regeneration [21].

There is an evidence of tissue alteration by MSCs through secretion of paracrine factors contained in extracellular vesicles (EVs). EVs are a group of cell-derived structures (composed of lipid bilayer membranes), which play an essential role in intercellular communication via transfer of bioactive proteins, lipids and RNAs and represent a potential source for circulating biomarkers of diseases [22]. EVs are generally divided, depending on their biogenesis, into subgroups, like exosomes (40–150 nm in diameters), microvesicles (150–1000 nm in diameter) and apoptotic bodies (50–2000 nm in diameter) [23]. Recent studies suggest possible substitution of the biological MSCs activity with MSC-derived exosomes [24–26]. Therefore, exosomes could represent a considerable alternative to cell therapy.

This review is focused on the characterization of MSCs-derived exosomes and their perspective using in cell-free therapeutic applications, as well as on the summarization of important facts about general MSCs´ paracrine secretion.

### Paracrine secretion of MSCs

MSCs perform their immunomodulatory activity not only through cell–cell interactions but also via strong paracrine impact. The MSCs´ paracrine effect was firstly described by Heynesworth et al. They notified secretion of a large spectrum of cytokines, chemokines and growth factors by MSCs with possible significant effects on cells in their periphery [27]. However, precise mechanism of action is still unknown and under examination. Numerous studies confirmed that factors secreted by MSCs could regenerate injured myocardium and improve cardiac function in porcine model [28], ameliorate acute renal failure and protect against limb tissue injury [29], promote in vitro and in vivo arteriogenesis [30] or support neovascularization [31].

One of the main pattern representing MSCs secretion of biological factors is by EVs which are classified as membrane vesicles filled with plenty of different proteins, microRNAs or/and messenger RNAs and have been progressively studied as the therapeutic agent in MSCs secretion [32]. The lipid bilayer of EVs encloses their bioactive capacity and protects them from enzymatic degradation. EVs are nowadays defined by their size, sedimentation rate, biogenesis pathway or protein delivery, but most of these parameters are neither terminal nor specific for any of EVs type. They have different structural and biochemical properties depending on their intracellular site of origin, which can affect their given functions [33]. Regardless of their origin, EVs are circular membrane particles possesing the characteristics of the origin cells, containing cytosol. In regard to their intracellular origin and the mechanisms of formation, EVs may be classified as exosomes, microvesicles and apoptotic bodies [23].

Apoptotic bodies are released as products of an appoptotic cell disassembly into subcellular fragments. There is an evidence that EVs generated during apoptosis have an important immunoregulatory role in autoimmunity, infection and cancer [34]. Microvesicles, also called as ectosomes or shedding vesicles, represent a heterogenous population formed by external budding and cleavage of the cell membrane. There is a large volume of phosphatidylserine on their surface and great number of proteins associated with lipid rafts (cholesterol-rich microdomains). Assembling of microvesicles is related to an increase of calcium ions which by calpain activation supports the cytoskeleton reorganization leading to the separation of plasma membrane protrusion from the cortical actin [35, 36]. Microvesicles may contain several plasma proteins depending on the type of the cell they originated and therefore specific markers are required for their identification. The generic marker is Anexin V. CD45 is used to identify leukocyte-derived microvesicles, CD42b/CD31− and CD62P for plateled-derived microvesicles, and CD31+ /CD42−, CD62E and CD144 are used for characterization of endothelial-derived microvesicles [37]. In addition, microvesicles may contain selectins, integrins, metalloproteinases and CD40 ligand [38]. On the other hand, exosomes are smaller and homogenous, have an endosomal origin and are formed by the internal budding of the multivesicular body membrane. The mechanism of their assembling and separation is still unknown [31]. Lipid bilayer of exosomes contains sphingomyelin, phosphatidylserine, phosphatidylcholine, phosphatidylethanolamine, phosphatidylinositol and monosialotetrahex-osylganglioside, which are similar to the cell plasma membrane composition [39]. Considered markers of exosomes are tetraspanins (CD9, CD63, CD81 and CD82), TSG101 (tumour susceptibility gene 101), heat shock proteins HSP70 and HSP90 and ALIX [39].

In general, it was shown that EVs are able to effectively copy the therapeutic effect of MSCs, mainly in tissue repair and regeneration in some preclinical models, e.g. exosomes potentially applied in wound healing and cutaneous regeneration [40], human adult liver stem cells—derived microvesicles increased hepatocyte proliferation associated with an accelerated morphological and functional recovery in a rat model [41] or human bone marrow MSCs—derived microvesicles increased proliferation and reduced apoptosis of tubular cells in a mice model [42].

### Exosomes

Presently, the best characterized EVs are exosomes, which secretion into extracellular area by hematopoietic cells, more specifically by reticulocytes, was firstly described in late 1980s [43–45]. Initially, exosomes secreted from cells were considered as homeostasis secondary products or cellular waste from cell injury without any significant influence on cells nearby. Nowadays, exosomes are considered as a special agent of intracellular communication, playing a major role in cellular processes including immune response [46], antigen presentation [47] and signal transduction [48]. It was indicated that exosomes are produced and released by various types of healthy cells involving adipocytes, epithelial cells, fibrolasts, neurons, astrocytes and Schwann cells. In addition, they were found in numerous types of body fluids including cerebrospinal, synovial and amniotic fluid, urine, sperm, saliva, blood, ascites, vitreous and brest milk [49].

### The biogenesis/formation and secretion of exosomes

**Fig. 2:**
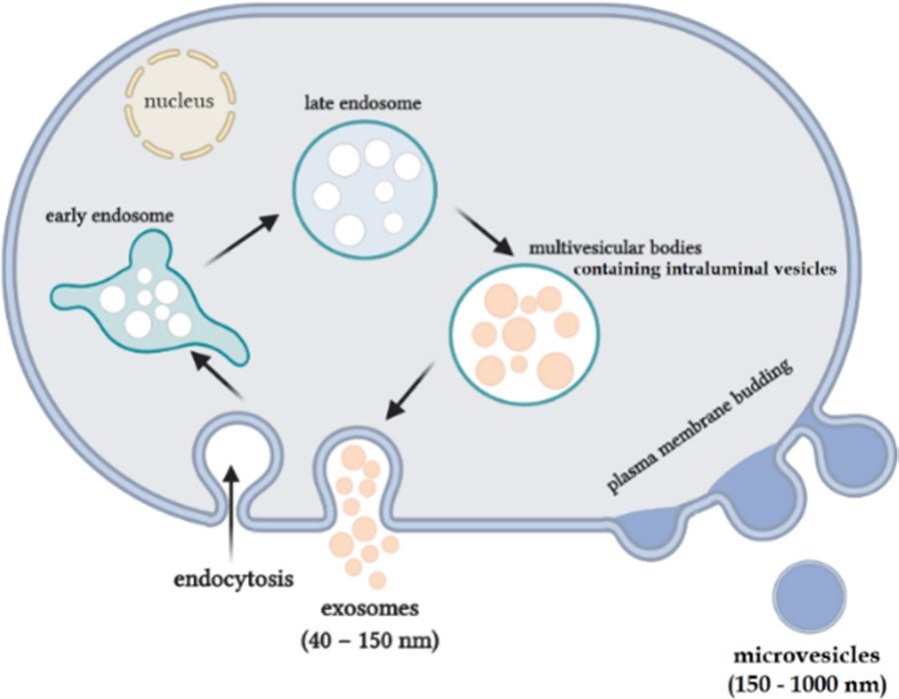
Schematic characterization of EVs (exosomes and microvesicles) formation. (Created with BioRender.com.)

In general, the biogenesis of exosomes begins within the endosomal system (Fig. 2) during which early endosomes (generated by internal budding) are unfolded into the late endosomes or multivesicular bodies (MVBs) and the endosomal membrane is invaginated to form intraluminal vesicles (ILVs) in the lumen of the organelles. The MVBs can either fuse with lysosomes to degrade their content or fuse with the plasma membrane to secrete the volume of ILVs as exosomes [50].

The endosomal sorting complex required for transport (ESCRT) machinery is very important for the MVBs/ILVs formation, vesicle budding and protein cargo sorting [51]. Ubiquitin is a relevant signal agent that transports membrane proteins and/or damaged cellular elements to lysosomes for degradation. It is also known as signal molecule for exosomal cargo sorting on the endosome membrane [52]. ESCRT machinery is composed from four multiprotein complexes, namely ESCRT-0, -I, -II, -III and the associated AAA ATPase VPS4 complex [51]. Separation of proteins to MVBs includes segregation of the ubiquitinated proteins into lipid rafts with ESCRT-0. TSG101 (ESCRT-I protein) is able to bind to ubiquitinated cargo proteins and sorts endocytic ubiquitinated cargos into MVBs. Subsequently, ESCRT-II complex is activated, which starts the oligomerization and production of the ESCRT-III complex. This complex is involved in proceeding of the budding process responsible for the sequestration of MVBs proteins, sends the deubiquitinating enzyme to remove the ubiquitin label from the cargo proteins and then sorts them into ILVs. Finally, ESCRT-III complex is separated from MVB membrane by sorting protein VPS4 and is unfolded by ATPase [53, 54]. The precise role of ESCRT machinery in the generating of ILVs secreted later as exosomes is still unclear. In the screening study of RNA interference targeting ESCRT associated proteins in HeLa cells was shown that the depletion of Hrs, TSG101 and STAM1 proteins can reduce the exosomes secretion [55]. It was examined by nanoparticle tracking analysis that knockdown of Hrs reduced exosome secretion from head and neck squamous cell carcinoma cells [56]. Likewise, exosome secretion was increased by knockdown of the ESCRT-III and associated proteins ALIX, VTA1, VPS4B and CHMP4C [55]. Specifically, increase of exosomal level and typical exosomal markers (CD63, HSP70) was confirmed after syndecan – syntenin – ALIX depletion in MCF-7 cells [57].

Alternatively, sorting of exosomal cargo into MVBs and following ILVs formation can occur via ESCRT independent mechanism. Proteolipid protein containing exosomes requires for their secretion ceramide which is able to initiate the exosome budding into MVBs [58]. Expression of tetraspanins (transmembrane proteins rich in exosomes) CD9 and CD82 increased the exosomal release of β-catenin (involved in regulation and organization of cell–cell adhesion and gene transcription) from HEK293 cells [59]. The oligomerization of oligomers could play a significant role in exosome biogenesis based on CD43 exosomal sorting in Jurkat T-cells [60]. Observably, there are various possible mechanisms for separation of bioactive molecules into exosomes, either ESCRT dependent or independent, allow to work depending on the cell type and/or cellular homeostasis. In addition, it was shown that numerous diseases and other pathological conditions enhance exosome secretion. Increased quantity of exosomes were noticed in tumor cells, by progression of inflammation, angiogenesis and coagulation [61–63].

### Molecular composition of exosomes

The molecular structure of exosomes is related not only to the cell type of origin but also to the microenvironment involving mechanical properties, biochemical impulses and topography, which could influence protein cargo regulation of the secreted exosomes [39]. Exosome secretion and their composition can also be modulated by other environmental factors such as oxygen level, type of disease, mechanical stress or media composition [64].

**Fig. 3:**
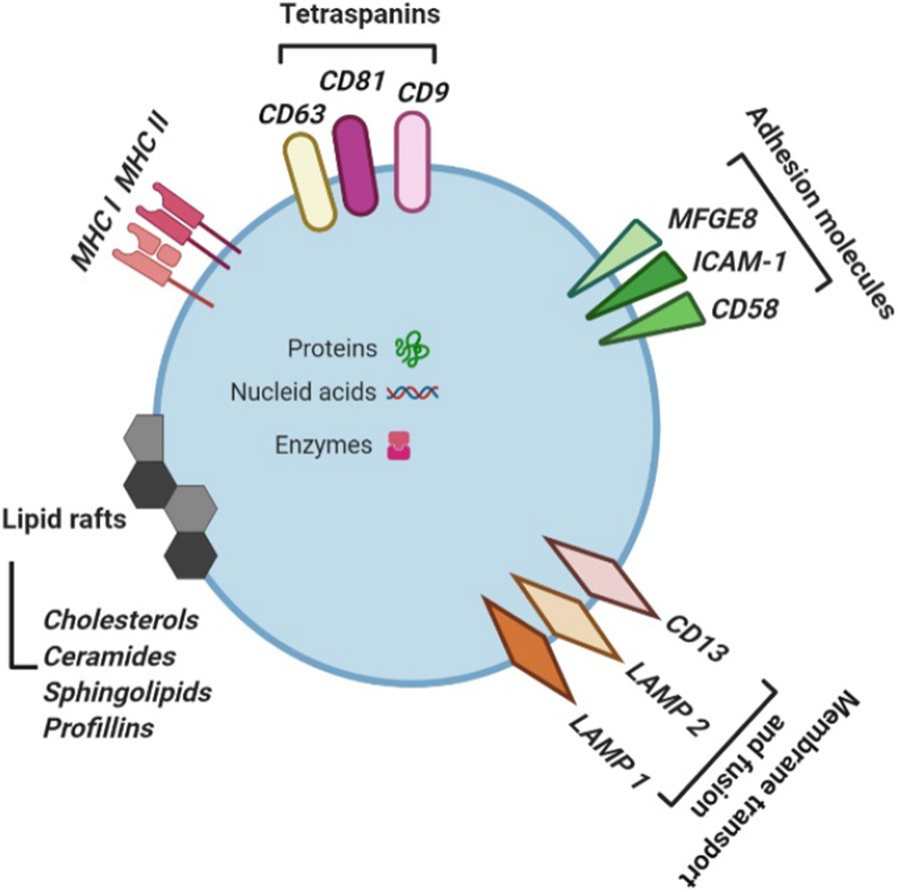
Exosome´s composition briefing: *MHC I, II*—major histocompatibility complex I, II); *MFGE8* -milk fat globule EGF factor 8 protein; *ICAM-1*—intercellular adhesion molecule 1; *LAMP 1, 2*—lysosomal-associated membrane protein 1, 2; proteins involved *heat shock proteins* (HSP60, HSP70, HSP90), *MVB biogenesis proteins* (Alix, TSG101, Ubiquitin, Clathrin), *cytoskeleton proteins* (profilin, cofilin, tubulin, actin, myosin, tropomyosin), *signaling proteins* (G protein, syntenin, MAPK, ERK ½, Rho); to enzymes belong pyruvate kinase, ATPase, PGK1, GADPH, aldolase, enolase; nucleic acids include mRNA, miRNA, siRNA, tRNA, DNA. (Created with BioRender.com.)

Exosomes are composed of various macromolecules involving unique lipid and protein structures and nucleic acids (Fig. 3). Exosomes are characterized by abundant amount of miRNAs with majority in the form of pre-miRNAs, which are inactive until their conversion to mature miRNAs [65]. Considering the endosomal origin of exosomes, they contain proteins participating in membrane transport and fusion (e.g. annexins, Rab, flotillin, GTPases), MVBs biogenesis (e.g. ALIX, TSG101) and also proteins associated with lipid microdomains (integrins and tetraspanins). Besides that, another frequently determinated proteins are associated with cytoskeleton (e.g. tubulin, myosin, actin) and metabolism (e.g. GADPH) [54], heat shock proteins (HSC70, HSC90), tissue specific proteins (e.g. MHC II located on the surface of exosomes secreted by dendritic cells or by B-lymphocytes) or proteins specific for cancer cell lines (e.g. glioma EGFR, breast cancer HER2, ovarian cancer CD24) [66].

Specifically, numerous studies on the protein and RNA composition of MSC-derived exosomes have been reported. Lai et al. investigated the proteome of HPLC-purified human embryonic stem cells—derived exosomes using mass spectrometry and cytokine array. They identified more than 850 proteins and detected total protein complement of a 20S proteasome with very high reliability [67]. Kang´s group realized proteomic analysis of the nanoscale size-based fractionation of exosomes from human neural stem cells and identified 103 proteins. Results from their study confirmed, that exosomes larger than ∼50 nm were morphologically different from those which were smaller than ∼50 nm [68]. MSC-derived exosomes were found to contain also all five enzymes involved in the ATP synthesis of glycolysis, namely glyceraldehyde 3-phosphate dehydrogenase, phosphoglycerate kinase, phosphoglucomutase, enolase and pyruvate kinase m2 isoform [69]. Furthermore, Arslan´s group detected enzymatically active CD73 in MSC-derived exosomes responsible for the generating of extracellular adenosine from released adenine nucleotides [69]. Exosomes are able to activate adenosine receptors and thus generate adenosine-affected phosphorylation of ERK1/2 and Akt in H9C2 cardiomyocytes [70].

The genetic information in RNAs of exosomes which are endocytosed by acceptor cells is allow to influence the protein expression in those cells. Exosomes contain RNAs mostly in size range less than 700 nt. Chen et al. identified the presence of small RNAs (less than 30 nt) in human embryonic stem cells-derived MSCs´ conditioned medium, which were encapsulated in cholesterol rich phospholipid vesicles [65]. Plethora of miRNAs responsible for post-transcriptional maintainig of gene expression were detected in MSC-derived exosomes which are active in acceptor cells [71] and participate in physiological and pathological processes. Research group of Ratajczak et al. reported that embryonic stem cells – derived exosomes are highly enriched in mRNA (for numerous transcription factors, receptors and cytokines) [72]. Furthermore, Valadi et al. identified different miRNAs including let-7, miR-1, miR-15, miR-16, miR-181 and miR-375 in exosomes isolated from mast-cell line (MC/9), primary bone marrow-derived mast cells (BMMC) and human mast-cell line (HMC-1) [73], which have been suggested to play an important role in exocytosis, tumorigenesis, angiogenesis and haematopoiesis [74]. Ono et al. reported that miR-23b promotes dormancy in breast cancer cells [75]. Exosomal miRNAs derived from umbilical cord MSCs, mainly represented by let-7f, miR-145, miR-199a and miR-221 supported the suppression of hepatitis C virus RNA replication [76]. Results of several sequencing studies also demonstrated, that exosomes isolated from human blood serum and urine contain marked amount of other RNA types, such as tRNA, rRNA, snRNA snoRNA, piRNA and scaRNA [77].

The current studies of the structure and composition of exosomes have relevant importance and are still under examination. Wang et al. compared paracrine functions in vivo and exosomal profiles of human endometrium-, bone marrow- and adipose-derived MSCs in a rat model of myocardial infarction. Analyses of exosomal microRNAs showed that miR-21 expression was improved in exosomes derived from endometrium [78], suggesting that innate differences of various MSC-derived exosomes have substantial influence on their clinical efficacy. The importance of exosomes has long been recognized also due to their capability to transfer important cellular cargoes (proteins, DNA, mRNA, miRNAs) to target cells. Recent evidences suggest that exosomes are involved both in normal physiological functions and in pathological conditions. Deeper understanding of the exosomes content may influence the study of various diseases. Some research groups demonstrated that tetraspanin complexes significantly contributes to selective target binding of exosomes to target cells [79, 80]. Thakur et al. showed that the presence of dsDNA in exosomes represented the whole genomic DNA and could be used for identification of mutations in parental tumor cells. They determined that tumor-derived exosomes carry dsDNA and may be use as a circulating biomarker in the early detection of cancer and metastasis [81]. Liang et al. used engineered exosomes for co-delivery of chemotherapeutic drug 5-fluorouracil and chemoresistance miR-21 inhibitor oligonucleotide to reduce the drug resistance in colorectal carcinoma and thus to improve the efficacy of cancer treatment [82]. Yang et al. demonstrated the capability of brain endothelial cell-derived exosomes to deliver siRNA across the brain-blood barrier in zebrafish and thus inhibit VEGF [83]. Results suggested potential application of natural exosome vesicles in the treatment of brain disease [83]. Raposo et al. showed that both human and murine B-lymphocytes secrete exosomes to induce antigen-specific MHC (major histocompatibility complex) II-restricted T cell responses, reffering to exosome usefulness as biological instruments in immunotherapy [84].

The therapeutic effect and biodistribution of exosomes is also greatly affected by the origin of exosome producing cells. MSC-derived exosomes regarding to their inner properties and source of origin may play a relevant role in their clinical efficiency and represent an ideal delivery system for intermediate processes in specific target cells.

### Therapeutic potential of MSC-derived exosomes

MSC-derived exosomes increasingly play an important role in intracellular communication mechanism and tissue repair and their clinical use may supply substantial advantages in comparison with their live cells due to potential to reduce undesirable side effects after application as well as infusional toxicities, uncontrolled cell growth and possible tumor formation. Moreover exosomes transplantation seems to be less risky and may have several advantages in contrast to cell applications. Exosomes are neither able to mutate and duplicate, nor induce metastasis. They have been tested in various animal models (Table 1) for human diseases (e.g. hypoxic pulmonary hypertension [85], acute kidney injury [86], liver fibrosis [87]) and it was detected that their functions are very similar to MSCs. First therapeutic potential of MSC-derived exosomes was described in a Langendorff heart model of acute myocardial ischemia/reperfusion injury in mice, where their cardioprotective effect was identified by myocardial infarct size reducing [88]. In this study, authors identified exosomes as cardioprotective elements in the MSCs´ paracrine secretion [88].

**Table 1:** List of therapeutic application of some MSC-derived exosomes from different sources in animal models of human disease

Tissue type	MSC origin	Method of exosome isolation	Exosome characterization	Disease focus	Animal type and model	Exosome administration	Related exosome cargo/pathway	Outcome	Refs
Bone marrow	Human	Centrifugation, ultracentrifugation	Determination of total protein concentration (BCA protein assay), TEM, NTA, western blotting (CD9, CD63, CD81, TSG101, and Alix markers)	Liver fibrosis	CCl_4_ induced liver fibrosis in SD rats	Injection through the tail vein	Wnt/β-catenin pathway	MSC-derived exosomes reduced liver fibrosis in vivo through the Wnt/β-catenin pathway Exosome treatment reduces the expression of PPARγ, Wnt3a, Wnt10b and β-catenin, what contributed to inhibition of downstream gene expression (WISP1, Cyclin D1) in both hepatic stellate cells and liver fibrosis tissue	[108]
	Human	Precipitation (ExoQuick exosome isolation)	Determination of total protein concentration (BCA protein assay), qNano nanopore-based detection, SDS-page (CD9, CD63, CD81), reversed-phase chromatography, Q Exactive mass spectrometry	Traumatic brain injury	Cortical impact Wistar rats model of traumatic brain injury	Intravenously via the tail vein	N.D	Exosomes derived from human BM-MSCs in 2D or 3D cultures improved functional recovery, promoted neurovascular remodeling and reduced neuroinflammation in rats after traumatic brain injury	[114]
	Human	Gradient ultracentrifugation, ultrafiltration	Determination of total protein concentration (BCA protein assay), electron microscopy, flow cytometry (CD63)	Bone defects	Calvarial defects in SD rats	Defects treated with hydgogel + EVs	miR-196a, miR-27a, miR-206	In vitro-EVs positively regulated expression of osteogenic genes and osteoblast differentiation In vivo-EVs stimulated bone formation in rats with calvarial defects	[119]
	Porcine	Ultrafiltration	Determination of total protein concentration (Bradford assay), NTA, flow cytometry (CD44 and CD90)	Synovitis	Porcine model of antigen-triggered synovitis	Intra-articular injections	N.D	Exosomes decreased synovial lymphocytes, the downregulated TNF-α transcripts and improved the impulse in exosome-treated joints	[116]
	Rat	Ultracentrifugation	Determination of total protein concentration (BCA protein assay), TEM, RT-PCR	Acute kidney injury	Acute kidney injury induced by gentamicin in Wistar rats	Injection into caudal vein	RNAase, RNA carried by the exosomes/microvesicles	BM conditioned media increased the renal function recovery. Protective effects were mediated by the exosome´ RNA in the conditioned media	[86]
	Rat	Differential centrifugation	Determination of total protein concentration (BCA protein assay), DLS, confocal microscopy, SEM, TEM, ELISA (CD9), flow cytometry (CD63), western blotting (CD81)	Acute liver injury	Ischemic/reperfusion liver injury and CCl_4_ induced acute liver injury in rats	Injection through hepatic portal vein	Exosome-rich fractionated secretome	In vitro – exosomes showed antiapoptotic and prosurvival effect, better HepG2 cells recovery and reduced cytotoxicity In vivo – exosomes improved liver regeneration and recovery from liver injury	[109]
	Rat	Precipitation (ExoQuick-TC exosome isolation)	Determination of total protein concentration (BCA protein assay), flow cytometry (CD63), TEM	Myocardial infarction	Acute myocardial infarction in SD rats	Intramyocardial injection	N.D	Exosomes improved cardiac function after ischemic injury In vitro – exosomes improved the tube formation of HUVEC cells and impaired T-cell function by cell proliferation inhibition In vivo – exosomes reduced infarct size and retained cardiac systolic and diastolic performance	[111]
	Rat	Ultracentrifugation	Electron microscopy	Myocardial infarction	SD rats myocardial ischemia/reperfusion model	Intramyocardial injection into the left ventricular wall	Autophagy machinery	Exosomes inhibited myocardial infarction pathogenesis, probably by autophagy regulation. Exosomes treatment suppressed the expression of Apaf1 (apoptotic protease activating factor 1) and increased the espression of ATG13 (autophagy-related protein13)	[112]
	Rat	Multistep centrifugation	Determination of total protein concentration (micro BCA protein assay)	Stroke	Middle cerebral artery occlusion Wistar rats model	Injection into the tail vein	N.D	Exosomes improved neurologic outcome by functional recovery and enhanced neurite remodeling, neurogenesis and angiogenesis. Exosomes systemic treatment improves neurologic outcome, significantly increased the synaptophysin immunoreactive area in ischemic boundary zone	[113]
	Mouse	Filtration, differential centrifugation, ultracentrifugation	Determination of total protein concentration (BCA protein assay), DLS, electron microscopy	Cardiac hypertrophy	Transverse aortic constriction mouse model	Intramyocardial injection	N.D	In vitro—exosomes inhibited cell hypertrophy stimulated with angiotensin II in cultured myocytes In vivo—exosomes significantly protected myocardium against cardiac hypertrophy, inhibited myocardial apoptosis and fibrosis and retained heart function when the pressure was overloaded	[110]
	Mouse (ischemic preconditioned)	Precipitation (ExoQuick exosome isolation)	Determination of total protein concentration (BCA protein assay), western blotting (CD9, CD63)	Alzheimer´s disease	Transgenic APP/PS1 mouse model	Injection through lateral caudal vein	miR-21, miR-181c	Exosomes improved memory functions and learning capabilities in mice. Hypoxic MSC-derived exosomes reduced effectively Aβ accumulation, increased the expression of synaptic proteins and enhanced the level of miR-21 in the brains of APP/PS1 mice	[115]
Umbilical cord	Human (Wharton’s jelly) and mouse BM	Precipitation, column size exclusion chromatography, ultracentrifugation	Electron microscopic analysis, determination of total protein concentration (Bradford assay) western blotting (CD63, ALIX, TSG101, CD81, CD9, hsp90, flotillin-1, Dicer), isolation and quantification of microRNAs	Hypoxic pulmonary hypertension	Hypoxia induced pulmonary hypertension in FVB strain mice	N.D	miR-204, miR-17	MSC-derived exosomes were able to inhibit pulmonary hypertension by inihition of hyperproliferative pathways, icluding suppression of the hypoxic activation of STAT3 signaling and the upregulation of the miR-17, whereas it increased lung levels of miR-204	[85]
	Human	Ultracentrifugation	Determination of total protein concentration (BCA protein assay), TEM, western blotting (CD9, CD81)	liver fibrosis	CCl_4_-induced liver fibrosis in mice	Injection into the left and right lobes of livers	(TGF)-b1/Smad signaling pathway	MSC-derived exosomes inhibited EMT and improved CCl_4_ induced liver fibrosis In vivo—exosome transplantation reduced TGF-β1 expression, inactivated Smad2 phosphorylation and inverted liver EMT In vitro – Exosome treatment of HL7702 cells after EMT caused reversed spindle-shaped cells and EMT associated marker expression	[87]
	Human	Precipitation (ExoQuick ULTRA EV isolation)	Electron microscopy, NTA, western blotting (TSG101, CD63, CD81), qPCR analysis, sequencing of miRNAs	Acute liver injury	CCl_4_-induced acute liver injury and endotoxemia in C57BL/6 mice	Injection	miR455-3p	Exosomes enriched in miR-455-3p were capable to inhibited the overactivation of monocyte/macrophages and reduced acute liver injury by inhibiting IL-6-related signaling pathways	[122]
	Human	Ultracentrifugation	TEM, western blotting (CD9, CD63, CD81)	Acute kidney injury	Cisplatin-induced acute kidney injury in SD rats	Renal capsule injection	p38MAPK pathway, ERK 1/2 pathway	Exosomes suppressed kidney injury and NRK-52E cell injury by improvement of oxidative stress and cell apoptosis and promotion of cell proliferation through activation of ERK1/2 in vivo and in vitro	[123]
	Human	Ultracentrifugation	Determination of total protein concentration (Bradford assay), TEM, flow cytometry	Acute kidney injury	Acute kidney injury model induced by ischemia–reperfusion injury in rats	Intravenous administration	N.D	Exosomes reduced cell apoptosis and improved proliferation 24 h after kidney injury, promoted angiogenesis by inducing VEGF elevation through HIF-1α independent manner	[124]
	Human	Differential centrifugation, ultracentrifugation	Determination of total protein concentration (BCA protein assay), western blotting (CD9, HSP70), TEM, NTA	Wound healing and angiogenesis	Skin burn wound model in rats	Subcutaneous injection	Wnt4	In vitro—exosomes elevated endothelial cell proliferation, migration and tube formation In vivo—exosomes improved angiogenesis in the repair of skin burn injury by delivering Wnt4 to activate Wnt/β-catenin signaling (tissue repair mechanism)	[125]
	Human	Ultracentrifugation	Determination of total protein concentration (BCA protein assay), TEM, western blotting (CD9, CD63, CD81, β-catenin, Wnt3a, β-actin)	Fracture healing	Model of femorale fracture in SD rats	Injection of the mix of hydrogel and exosomes into the fracture	N.D	Exosomes participated in the repair of fracture in rats through the Wnt signaling pathway by increasing of β-catenin and Wnt3a protein expressions	[126]
	Human	Ultracentrifugation	Determination of total protein concentration (BCA protein assay), NTA, western blotting (CD81)	Wound healing	Skin-defect model in ICR and BALB/c-υ mice	Injection of the mix of hydrogel and exosomes around the wound	miR-21, miR-23a, miR-125b, miR-145	Exosomes enriched in specific microRNAs (miR-21, -23a, -125b, and -145) inhibited myofibroblast formation, inhibited TGF-β2, TGF-βR2 and SMAD2 pathway and accordingly suppressed the expression of α-SMA gene and reduced collagen I deposition. Significant role of exosomes for anti-scarring ability and the myofibroblast-suppressing was showed both in vitro and in vivo by blocking miRNAs inside the exosomes	[127]
	Human	Ultracentrifugation	TEM, determination of total protein concentration (BCA protein assay), NTA, western blotting (CD9, CD63, CD81)	Inflammatory bowel disease	DSS-induced inflammatory bowel disease mouse model	Injection through the tail vein	N.D	Exosomes could improve inflammatory bowel disease In vitro – coculture with exosomes suppressed the expression of iNOS and IL-7 in mouse enterocelia macrophages In vivo – exosomes reduced the expression of pro-inflammatory cytokines TNF-α, IL-1β, IL-6) and increased the expression of anti-inflammatory cytokine (IL10)	[128]
	Human	Ultracentrifugation	Determination of total protein concentration, TEM, Zetasizer, western blotting (CD9, CD63, CD81)	Colitis	Colitis induction in C57BL/6 mice	Intraperitoneal injection	N.D	In vitro – exosomes decreased pro-inflammatory cytokines (IFN-γ, TNF-α, IL-1β) concentration and enhanced the secretion of anti-inflammatory cytokines (TGF-β1, IL-10) In vivo – exosomes showed therapeutic activity in experimental colitis via suppressing inflammation machinery, improved clinical symptoms and histological severity	[90]
Adipose	Human	Ultracentrifugation	Determination of total protein concentration (Bradford assay), TEM, NTA, SIOS,	Alzheimer’s disease	N.D	N.D	Neprilysin	Exosomes secrete enzymatically active neprilysin. Transfer of exosomes to N2a cells significantly decreased both the intracellular and extracellular Aβ40 and Aβ42 levels	[133]
	Pig	Ultracentrifugation	NTA, TEM, western blotting (CD9, CD29, CD63), RT PCR (mRNA content of IL-10)	Renal inflammation	Metabolic syndrome and renal artery stenosis model in domestic pigs	Intrarenal delivery	IL-10	Exosomes reduced renal inflammation, enhanced the reparative macrophages number and increased expression of IL-10. Exosomes were able to reduce renal fibrosis and to improve stenotic kidney function	[130]
	Rat	Ultrafiltration, ultracentrifugation	Determination of total protein concentration (BCA protein assay), TEM, western blotting (CD9, CD63, HSP70, CD81)	Ischemic heart disease	Myocardial ischemia/reperfusion model in SD rats	Infusion through the tail vein	N.D	Exosomes protected ischemic myocardium from ischemia/reperfusion injury through the Wnt/β-catenin signaling pathway activation In vitro – exosomes reduced cell apoptosis and the expression of Bax, improved cell viability and the expression of Bcl-2 and Cyclin D1 in hypoxia/reoxygenation-induced H9c2 cells In vivo – exosomes significantly reduced ischemia/reperfusion-induced myocardial infarction, it was showed decrease in serum levels of creatine kinase-myocardial band, lactate dehydrogenase, and cardiac troponin I. After exosome treatment was observed attenuation of ischemia/reperfusion-induced apoptosis accompanied by the increase of Bcl-2 and decrease of Bax, and inhibition of Caspase 3 activity in rat myocardium	[131]
	Mouse	Precipitation and magnetic beads purification (MagCapture Exosome Isolation)	Determination of total protein concentration (BCA protein assay), NTA, TEM, western blotting (CD29, CD63)	Acute myocardial infarction	N.D	N.D	N.D	Exosomes reduced apoptosis in myocardial cells subjected to oxidative stress in vitro	(132)

N.D, not defined in the given reference; TEM, transmission electron microscopy; NTA, nanoparticle tracking analysis; SIOS, scanning ion occlusion sensing; EMT, epithelial-mesenchymal transition; DLS, Dynamic light scattering; SEM, scanning electron microscopy

Several preclinical studies compared the beneficial effects of cell therapy based on MSCs and cell-free therapy based on MSC-derived EVs/exosomes and showed that they had similar therapeutic outcomes. Comparative analyses of MSCs and their EVs demonstrated different genetic cargo and protein content that play a significant role in biological processes, including angiogenesis, adipogenesis, apoptosis, regulation of inflammation, blood coagulation and extracellular matrix remodeling. Application of mice adipose MSCs in comparison with its conditioned medium had the same effect on sympotms of chronic colitis mouse model. Clinical symptoms and tissue damages were suppressed in treated mice [89]. Zhi et al. indicated that the application of umbilical cord MSC-derived exosomes (200 µg) resulted in amelioration of clinical symptoms, reduction of colonic damage and decrease of the inflammatory state in mice colitis when compared with MSCs (1 × 10^6^ cells) administration [90]. Shao et al. compared activity of rat bone marrow MSCs and MSC-derived exosomes in a rat acute myocardial infarction model. It was showed a superior beneficial effects of MSC-derived exosomes in contrast to MSCs in cardiac repair. There were observed differences in expression profiles of several miRNAs from that of MSCs detected through miRNA sequence analysis [91]. A recent cutaneous wound model study in rabbits reported that intradermal injection of EVs derived from adipose and bone marrow MSCs were superior to MSCs injection in vivo. Furthermore, adipose MSC-derived EVs enhanced wound healing better than EVs from bone marrow [92]. In the study by Gatti et al. intravenous administration of human bone marrow MSC-derived EVs had the same efficacy as MSCs on the treatment of acute kidney injury in rats by inhibiting apoptosis and stimulating tubular cell proliferation [93]. In an induced experimental autoimmune encephalomyelitis murine model of multiple sclerosis, both human placental MSCs and its MSC-derived EVs showed regenerative effects and prevented oligodendroglia degradation and demyelination [94]. Another preclinical study showed that MSC-derived exosomes could be a promising cell-free therapeutic strategy for the treatment of Alzheimer’s disease. It was demonstrated that 28 days after intervention of mice groups with 10 μg exosomes and 1 × 10^6^ MSCs separately had similar beneficial effects in improvement of neurogenesis and cognitive functions [95].

From the preclinical studies of MSC-derived exosomes therapy to the clinical application, many critical parameters should be resolved and determined, including clarification of important factors and conditions, defining optimal MSC culture conditions and protocols for precise monitoring of exosome formation, isolation, its characterization and storage. The biological effect of MCS-derived exosomes is mainly affected by the source of MSCs. The ideal source would be a high-exosome-yielding cell with a high expansion capacity [96, 97]. Further relevant requirement is the age of the donor tissue considering the exosome production might be indirectly connected with mentioned factor. Isolated exosomes are routinely identified by vesicle size and expression of typically tetraspanin markers CD63, CD9 and CD81. Production of exosomes could be enhanced by changing of several cell cultivation conditions, like increasing of intracellular calcium, or serum starvation. The long lasting donor HEK293 cell cultivation and maintaining cells at acidic pH could results in considerably increased production of exosomes [98]. Pre-conditioning of MSCs with hypoxia [99, 100], cytokines [101, 102] and another biomoleculs or chemicals (e.g. LPS [103], thrombin [104], NO [105], H_2_O_2_ [106]) also evoked the increase of exosomes activity, directly or indirectly by increasing MSCs function. Further important requirements for exosome preservation is an adequate storage. Sokolova et al. detected that the exosomes diameter decreased within 4 days at 4 °C and 2 days at 37 °C, indicating a structural change or degradation of exosomes, but storage at − 20 °C did not affect their size [107]. Extensive questions concerning of clinical grade exosomes production in sufficient quantity and of influence of different strategies on exosome potency are still under examination.

## Bone marrow MSC-derived exosomes

### Improvement of liver regeneration by BM MSC-derived exosomes

The potential of bone marrow (BM) MSC-derived exosomes for the treatment of various disease pathologies seems to be obvious. Rong et al. demonstrated the ability of human BM MSC-derived exosomes to reduce liver fibrosis in a carbon tetrachloride (CCl_4_)-induced liver fibrosis model of Sprague Dawley (SD) rats through the Wnt/β-catenin pathway. They also indicated the recovery of markers related to improved liver features, increasing hepatocyte regeneration and inhibition of inflammation process (significantly decreased inflammatory cytokines) [108]. Damania et al. studied the capability of rat BM MSC-derived exosomes present in fractionated MSC secretome to reduce liver injury in vitro in both 2D and 3D culture conditions of HepG2 cells and in in vivo rat models of acute liver injury caused by CCl_4_. Anti-apoptotic, anti-oxidative and prosurvival effects were shown in in vitro models of liver injury. In addition, the exosome rich fraction of conditioned media improved liver regeneration and recovery in vivo [109].

### Cardioprotection by BM MSC-derived exosomes

The multiple therapeutic effects of BM MSC-derived exosomes have also been detected in cardiovascular, ischemic and reperfusion diseases. Currently, Chen et al. established significant protection of myocardium against hypertrophy, inhibition of myocardial apoptosis and reduction of cardiac fibrosis by using mice BM MSC-derived exosomes in the murine pressure overload induced cardiac hypertrophy model [110]. Teng et al. in their study hypothesized about a significant role of rat BM MSC-derived exosomes in the cardioprotection through angiogenesis and anti-inflammation in SD rats with acute myocardial infarction. They shown an efficacious action of exosomes in cardiac remodeling post-myocardial infarction in vivo. Accordingly, obtained results indicated that exosomes supported angiogesesis in vitro in human umbilical vein endothelial cell line (HUVEC). Furthermore, the proliferation of CD3 stimulated T-cells was reduced after exosome treatment, which means decrease in proliferation of spleen lymphocytes [111]. The rat myoblast cell line H9c2 was used to study myocardial pathogenic processes as cellular hypoxia‑reoxygenation model. Inhibition effect of cell proliferation, migration and also of suppresion of cardiomyocyte apoptosis during hypoxia-reoxygenation was revealed after rat BM MSC-derived exosome treatment [112]. In addition, quantity of both apoptosis- and autophagy-competent functional proteins and Apaf1 (apoptotic protease activating factor 1) and ATG13 (autophagy-related protein 13) gene expression in these treated H9c2 cells exhibited modulations in accordance with SD rat myocardial ischemia/ reperfusion model. Apaf1 expression was considerably suppressed and ATG13 expression was significantly increased in vivo after exosome treatment. Authors concluded, that myocardial injury associated with myocardial infarction could be inhibited with BM MSC-derived exosomes, alternatively throught regulation of autophagy mechanism [112].

### BM MSC-derived exosomes and recovery after stroke and traumatic brain injury

In a stroke model (middle cerebral artery occlusion model) in Wistar rats, Xin et al. indicated that systemic administration of rat BM MSC-derived exosomes significantly enhanced functional recovery and improved neurite remodeling, neurogenesis and angiogenesis [113]. Therefore, exosomes could be effectively used for stroke treatment. Zhang et al. used human BM MSC-derived exosomes for the treatment of experimental traumatic brain injury in controlled cortical impact model in Wistar rats. Similarly, the improvement of functional recovery and promotion of neurovascular remodeling were demonstrated [114]. Administration of BM MSC-derived exosomes could regenerate cognition functions and memory impairment in neurological and neurodegenerative diseases. Exosomes derived from MSCs preconditioned by hypoxia supressed amyloid β accumulation and enhanced the synaptic protein expression in the brains of transgenic APP/PS1 mice (Alzheimer´s disease mice). Furthermore, reduced activation of astrocytes and microglia and changes in levels of inflammatory factors (increase of anti-inflammatoty cytokines IL-4, IL-10 and decrease of pro-inflammatory cytokines TNFα and IL-1β) were observed [115].

### Anti-inflammation mediated by BM MSC-derived exosomes

Another promising therapeutic feature of porcine BM MSC-derived exosomes was evaluated by Casado et al. They showed the anti-inflammatory effect of exosomes in porcine model (large white pigs) of antigen-triggered synovitis. The local inflammation in animals caused by intra-articular injection of BSA leads to an elevated level of white blood cells in synovial fluid. Interestingly, there were found no differences of white blood cells in joints after exosome administration, but significant decrease in the lymphocytes accompanied by a noteworthy decline of only one (TNFα) from eight tested inflammatory cytokines in synovial fluid was revealed [116]. It is interesting, that TNFα antagonists (e.g. infliximab, golimumab, etanercept) are generally used for the treatment of rheumatoid arthritis [117].

### Influence of BM MSC-derived exosomes on bone regeneration

Osteogenically differentiated human BM MSCs and subsequently derived EVs were used in the study of Martins et al.. They demonstrated, that human BM MSC-derived vesicles have osteoinductive potential characterized by early activation of alkaline phosphatase, early overexpression of the activator bone morphogenetic protein 2, transient increase in expression of Sp7 transcription factor (osterix) and secretion of phosphoprotein 1 (osteopontin) and integrin-binding sialoprotein (bone sialoprotein) [118]. Qin et al. tested the BM MSC-derived exosomes in the regulation of osteoblast activity in vitro and bone regeneration in vivo. Osteoblasts treated with miR-196a exhibited the best osteogenic activity in comparison with miR-27a and miR-206 treatment. Mentioned miRNAs are typical osteogenic related RNAs and are highly enriched in BM MSC-derived exosomes [119]. They also generated calvarial bone defects in SD rats and then applied hydrogel with EVs, which resulted in accelerated bone regeneration and indicated obvious improvement of the defect repair in comparison with hydrogel without EVs [119]. Narayanan et al. confirmed that exosomes from osteogenic human marrow MSCs are able to trigger lineage-specific differentiation of undifferentiated human BM MSCs [120]. Moreover, Shimbo et al. showed that the introduction of synthetic miR-143 into BM MSCs leads to an increase in not only the extracellular miR-143 but also increased secretion of exosomes. Such exosome-formed miR-143 was transferred to human osteosarcoma cell line 143B and caused suppression of their migration. It seems, that BM MSC-derived exosomes are also able to act as effective delivery system [121].

## Umbilical cord MSC-derived exosomes

### Reduction of liver fibrosis and liver injury by UC MSC-derived exosomes

Umbilical cord (UC) MSCs and their exosomes have also extensive potential in regenerative medicine, but their fundamental mechanism of action is still unknown. Li et al. used UC MSC-derived exosomes to treat CCl_4_-induced mouse liver fibrosis on Kunmingbai strains mice. It was shown that transplantation of human UC MSC-derived exosomes caused successful decrease of the serum fibrotic marker hyaluronic acid, TGF-β1 and serum aspartate aminotransferase and reduced hepatic inflammation and collagen deposition. Entire improvement after liver injury was confirmed [87]. Likewise, Jiang et al. identified hepatoprotective activities of human UC MSC-derived exosomes throught antioxidant defenses in mouse models (BALB/c female mice) of acute and chronic liver injury and liver tumor induced by CCl_4_ injection. They detected suppression of the liver tumor development, inhibition of oxidative stress in liver tumor, reduction of oxidative stress, inhibition of apoptosis in liver fibrosis and accordingly, reduction of oxidative stress and inhibition of apoptosis in acute liver injury after human UC MSC-derived exosome administration [71]. Shao et al. described large production of miR-455-3p enriched exosomes by human UC MSCs and their ability to suppress macrofage activation and reduce acute liver injury in mice model by inhibiting IL-6 signaling pathway [122].

### Influence of UC MSC-derived exosomes on treatment of kidney injury

Furthermore, Zhou et al. studied the influence of human UC MSC-derived exosomes in SD rat model of kidney injury induced by cisplatin and in rat NRK-52E cells treated with or without cisplatin in vitro. There was indicated activation of the p38MAPK pathway followed by the increase of caspase 3 in NRK-52E cells after cisplatina treatment. Increase of apoptotic cells and oxidative stress were also observed. By contrast, these parameters were significantly reduced after human UC MSC-derived exosome administration. Accordingly, human UC MSC-derived exosomes moderated tubular oxidative damage, suppressed renal cell apoptosis and promoted renal cell proliferation in vivo in rats [123]. The major reason of acute kidney injury is ischemia/reperfusion injury in hospitalized patients. Therefore, Zou et al. in other study showed, that a single intravenous administration of human UC MSC-derived exosomes in rats with acute kidney injury induced by ischemia/reperfusion injury elevated renal capillary vessel density and alleviated renal fibrosis by increase of proangiogenic vascular endothelial growth factor (VEGF). In this process, reduction of HIF-1α (hypoxia inducible factor) was also observed. Exosomes were able to reduce cell apoptosis and improve proliferation after kidney injury [124].

### Enhancement of fracture healing and wound healing by UC MSC-derived exosomes

Zhang et al. demonstrated intensive support of cutaneous wound healing and angiogenesis in vivo in a rat model of skin-deep second degree burn wound by human UC MSC-derived exosomes [125]. The Wnt signaling pathway plays an important role in angiogenesis mediated with the endothelial cell proliferation modulation, migration, vascular sprouting and remodeling, and vascular system maturation. UC-MSC-derived exosomes promote the tube formation, proliferation and migration of endothelial cells in vitro. In addition to that, applied exosomes improved angiogenesis by delivering Wnt4 to activation of Wnt/β-catenin in endothelial cells which could be one of the possible mechanism for tissue repair [125]. Likewise, Zhou et al. investigated the role of human UC MSC-derived exosomes in the Wnt signaling and their influence on femoral fracture healing in SD rats. Increase of β-catenin and Wnt3 expression indicating presumed participation of injected exosomes in repairing of the fracture was identified [126]. An important knowledge in this area is the study of Fang et al., in which they found that UC MSC-derived exosomes, especially exosomal microRNAs, decreased scar formation and myofibroblast accumulation in a skin-defect ICR mouse (Swiss-Hauschka mice) and nude mouse (BALB/c-υ) model. Myofibroblast formation can generally result in abnormal scarring. It was shown, that specific exosomal microRNAs (miR-21, miR-23a, miR-125b, and miR-145) inhibited redundant α-smooth muscle actin (α-SMA) and collagen I deposition and also suppressed TGF-β/SMAD2 signaling pathway [127].

### UC MSC-derived exosomes relieve bowel diseases

UC MSC-derived exosomes have high potential in the treatment of inflammatory bowel diseases involving chronic inflammation of the gastrointestinal tract, both Crohn´s disease and ulcerative colitis in the future. Mao et al. demonstrated decrease of pro-inflammatory cytokines IL-6, IL-1β, TNF-α expression and increase of anti-inflammatory cytokine IL-10 expression after UC MSC-derived exosomes treatment in inflammatory bowel disease in a mice model. Interestingly, significant inhibition of IL-7 expression was also observed in the colon mucosa tissues and spleens in a mice model [128]. The serum cytokine level of IL-7 is normally increased in inflammatory bowel disease patients [129]. Similarly, single intraperitoneal injection of UC MSC-derived exosomes resulted in a significant reduction of the clinical symptoms and colonic damages in the mouse model of dextran sodium sulfate-induced colitis through suppression of inflammation mechanism [90].

## Adipose MSC-derived exosomes

### Attenuation of kidney inflammation by AD MSC-derived exosomes

Adipose (AD) MSC-derived exosomes as well as exosomes derived from BM and UC MSCs present a multipotent and rich therapeutic role in the improvement of the injury repair of many tissues. AD MSC-derived exosomes are more abundant and have lower risk of side effects. A single intrarenal delivery of pig AD MSC-derived exosomes in a porcine model of metabolic syndrome and renal artery stenosis resulted in reduction of renal inflammation, enhancement of the reparative macrophages number and elevation of anti-inflammatory cytokine IL-10 expression. Furthermore, exosome administration lowered renal vein level of pro-inflammatory cytokines TNF-α, IL-1β and IL-6 [130]. Results in the study of Eirin et al., established attenuation of renal fibrosis and improvement of stenotic kidney function after AD MSC-derived exosome treatment [130].

### Cardioprotection by AD MSC-derived exosomes

It was observed, that AD MSC-derived exosomes are able to protect myocardium against acute ischemia/reperfusion induced necrosis and apoptosis in SD rat myocardial ischemia/reperfusion model. Ischemia/reperfusion injury in rats was accompanied with a remarkable decrease of Bcl-2 and an obvious increase in Bax expression. Both were eliminated after exosome administration. It was also observed, that AD MSC-derived exosomes attenuated hypoxia/reoxygenation induced apoptosis and promoted cell survival in H9c2 cell line [131]. In addition, Cui et al. hypothesized that AD MSC-derived exosome administration could protect ischemic myocardium through activation of Wnt/β-catenin signaling in vivo [131]. Liu et al. determined the protective influence of mouse AD MSC-derived exosomes on cardiomyocytes under oxidative stress in vitro [132].

### Potential of AD MSC-derived exosomes for Alzheimer´s disease treatment

Interestingly, Katsuda et al. demonstrated remarkable potential of AD MSC-derived exosomes for Alzheimer´s disease therapy [133]. They showed that AD MSC-derived exosomes exhibited neprilysin specific enzyme activity. Neprilysin is the most essential enzyme that degrade amyloid beta peptide in the brain. In addition, transfer of mentioned exosomes to neuroblastoma N2a cells resulted in a decrease of both intracellular and extracellular amyloid beta peptide grades, suggesting a promising therapeutic approach for exosome-based Alzheimer´s disease treatment [133].

### Role of AD MSC-derived exosomes in tumor progression

Recently, the influence of MSC-derived exosomes on tumor progression in both inhibiting and supporting mode was intensively described. Reza et al. indicated that human AD MSC-derived exosomal miRNAs have significant inhibitory influence on the regulation of different ovarian cancer cells [134]. Exosomes collected from human AD MSC-derived conditioned medium inhibited the growth and proliferation of ovarian cancer cells A2780 and SKOV-3. Decreased cell viability and wound healing of cancer cells were also observed after exosome treatment. Furthermore, collected exosomes caused apoptosis by increasing of pro-apoptotic signalling molecules Bax, caspase 3 and caspase 9 and by decreasing of anti-apoptotic bcl-2 protein [134].

### Clinical perspectives

Clinical applications using exosome technology as cell-free therapy has become an important field of research over the last years. Currently, 91 clinical trials involving exosomes are listed on www.clinicaltrials.gov. Exosomes used in these trials are mainly derived from several body fluids and are used as early diagnostic tools in prediction of various diseases.

The clinical use of human MSC-derived exosomes is limited due to rigorous resolution of critical parameters involved in the translation process of preclinical studies to the clinical ones. These paramaters include the optimal MSC culture conditions and protocols for exosome production, isolation, and storage with a considerable effect on the uniformity of optimal dose, exosome administration and efficacy evaluation [2, 24]. Various approaches to optimize the therapeutic efficacy of exosomes are being developed. In general, the substantial requirement is a standardization of the classification and extraction method of exosomes from various body fluids, including definition of using of lower biofluid volume, higher purity and yield. Also the identification and better characterization of specific EVs subgroups is needed because different EVs could involve different biological effects. Whereas actual extraction methods of exosomes are too diverse for confirmation of its purity, it is necessary to standardize the protocols and characterization methods before application of exosomes in clinical trials. In addition, determination of the optimal dose, adequate time and appropriate method for exosome administration with maximal targeted efficacy, biological safety and without adverse effects must be confirmed before their clinical use.

Up to date, there are 15 clinical trials related to MSC-derived exosomes, registered on Clinicaltrials.gov, which are summarized in Table 2. Some of these studies have been completed or are recruiting/about to open to accrual. The trial NCT04491240 is focused on the evaluation of safe and effective method of MSC-derived exosomes aerosol inhalation in SARS-CoV-2 associated pneumonia and is only one trial which has been posted the results. Similar issue is performed in the completed pilot clinical trial NCT04276987 where the safety and efficiency of allogenic AD MSC-derived exosomes inhalation in the treatment of patients hospitalized with new coronavirus pneumonia is investigated. In completed trial NCT03562715 peripheral blood exosomes´ miRNA136, miRNA494 and miRNA495 genes expression in comparison to UC MSC conditioned media exosomes in patients with pre-eclampsia (pregnancy complication) was indentified. Based on the received and published data, MSC-derived exosomes are going to be great biological tools for diabetes, stroke, Alzheimer´s Disease and cancer therapy. Actually, it is hopeful to delve deeper into the potential of MSC-exosomes among SARS-CoV-2 pneumonia therapy and provide effective treatments with the highest safety.

**Table 2:** List of clinical trials of MSC-derived exosomes-based therapies (source: www.clinicaltrials.gov)

NCT number	Study title	Study start	Condition	Intervention	Phase	Status
NCT04602104	A Clinical Study of Mesenchymal Stem Cell Exosomes Nebulizer for the Treatment of ARDS	October 2020	Acute Respiratory Distress Syndrome	Allogenic human MSC-Exos	Phase 1 Phase 2	Not yet recruiting
NCT04602442	Safety and Efficiency of Method of Exosome Inhalation in COVID-19 Associated Pneumonia (COVID-19EXO2)	October 1, 2020	Covid19; SARS-CoV-2 PNEUMONIA	MSC-Exos	Phase 2	Enrolling by invitation
NCT04173650	MSC EVs in Dystrophic Epidermolysis Bullosa	September 2020	Dystrophic Epidermolysis Bullosa	AGLE 102	Phase 1 Phase 2	Not yet recruiting
NCT04356300	Exosome of Mesenchymal Stem Cells for Multiple Organ Dysfuntion Syndrome After Surgical Repaire of Acute Type A Aortic Dissection	September 1, 2020	Multiple Organ Failure	UC MSC-Exos	Not Applicable	Not yet recruiting
NCT04491240	Evaluation of Safety and Efficiency of Method of Exosome Inhalation in SARS-CoV-2 Associated Pneumonia	July 20, 2020	Covid19; SARS-CoV-2 PNEUMONIA	MSC-Exos	Phase 1 Phase 2	Completed has results
NCT04544215	A Clinical Study of Mesenchymal Progenitor Cell Exosomes Nebulizer for the Treatment of Pulmonary Infection	July 1, 2020	Drug-resistant	Human AD MS progenitor cell-Exos	Phase 1 Phase 2	Recruiting
NCT04388982	the Safety and the Efficacy Evaluation of Allogenic Adipose MSC-Exos in Patients With Alzheimer's Disease	July 1, 2020	Alzheimer´s Disease	allogenic AD MSC-Exos	Phase 1 Phase 2	Recruiting
NCT03608631	iExosomes in Treating Participants With Metastatic Pancreas Cancer With KrasG12D Mutation	March 2020	Pancreatic cancer with KrasG12D mutation; Metastatic Pancreatic Adenocarcinoma; Pancreatic Ductal Adenocarcinoma; Stage IV Pancreatic Cancer AJCC v8	MSC-Exos with KRAS G12D siRNA	Phase 1	Not yet recruiting
NCT04313647	A Tolerance Clinical Study on Aerosol Inhalation of Mesenchymal Stem Cells Exosomes In Healthy Volunteers	March 16, 2020	Healthy	Allogenic AD MSC-Exos	Phase 1	Recruiting
NCT04213248	Effect of UMSCs Derived Exosomes on Dry Eye in Patients With cGVHD	February 18, 2020	Dry Eye	UC MSC-Exos	Phase 1 Phase 2	Recruiting
NCT04276987	A Pilot Clinical Study on Inhalation of Mesenchymal Stem Cells Exosomes Treating Severe Novel Coronavirus Pneumonia	February 15, 2020	Coronavirus	Allogenic AD MSC-Exos	Phase 1	Completed
NCT03384433	Allogenic Mesenchymal Stem Cell Derived Exosome in Patients With Acute Ischemic Stroke	April 17, 2019	Cerebrovascular Disorders	Allogenic MSC-Exos enriched by miR-124	Phase 1 Phase 2	Recruiting
NCT03437759	MSC-Exos Promote Healing of MHs	March 1, 2017	Macular Holes	MSC-Exos	Early Phase 1	Recruiting
NCT03562715	microRNAs Role in Pre-eclampsia Diagnosis	November 28, 2016	Pre-eclampsia			Completed
NCT02138331	Effect of Microvesicles and Exosomes Therapy on β-cell Mass in Type I Diabetes Mellitus (T1DM)	April 2014	Diabetes Mellitus Type 1	UC blood MSC-Exos	Phase 2 Phase 3	Unknown

NCT number, ClinicalTrials.gov identifier; MSC-Exos, mesenchymal stem cells-derived exosomes

## Conclusion

MSCs mainly exert their therapeutic effects through the secretion of paracrine factors to reduce inflammation, cellular injury and enhance cell and tissue repair. MSC-derived exosomes probably work in a similar manner and have the capacity to interact with multiple cell types, enabling the cells to recover, repair and regenerate within the tissue. Due to their ability to deliver genetic material, immunomodulatory proteins, enzymes, and growth factors directly to the recipient cells, they also represent an ideal multifunctional delivery system. MSC-derived exosome therapy may be an emerging and a promising tool for the treatment of various diseases, mainly of those with an inflammatory component. Whats more, encouraging results of preclinical and clinical data predicted that MSC-derived exosome treatment could be superior to cell-based therapy in the meaning of safety and versatility.

## Data Availability

Not applicable.

## Abbreviations

MSCs: Mesenchymal stem cells
EVs: Extracellular vesicles
MVBs: Multivesicular bodies
ILVs: Intraluminal vesicles
BM: Bone marrow
UC: Umbilical cord
AD: Adipose
ESCRT: The endosomal sorting complex required for transport
SD rats: Sprague Dawley rats
